# Functional analysis of polymorphisms at the S1/S2 site of SARS-CoV-2 spike protein

**DOI:** 10.1371/journal.pone.0265453

**Published:** 2022-03-25

**Authors:** Prerna Arora, Anzhalika Sidarovich, Luise Graichen, Bojan Hörnich, Alexander Hahn, Markus Hoffmann, Stefan Pöhlmann

**Affiliations:** 1 Infection Biology Unit, German Primate Center, Göttingen, Germany; 2 Faculty of Biology and Psychology, Georg-August-University Göttingen, Göttingen, Germany; 3 Junior Research Group Herpesviruses - Infection Biology Unit, German Primate Center, Göttingen, Germany; Stanford University, UNITED STATES

## Abstract

Several SARS-CoV-2 variants emerged that harbor mutations in the surface unit of the viral spike (S) protein that enhance infectivity and transmissibility. Here, we analyzed whether ten naturally-occurring mutations found within the extended loop harboring the S1/S2 cleavage site of the S protein, a determinant of SARS-CoV-2 cell tropism and pathogenicity, impact S protein processing and function. None of the mutations increased but several decreased S protein cleavage at the S1/S2 site, including S686G and P681H, the latter of which is found in variants of concern B.1.1.7 (Alpha variant) and B.1.1.529 (Omicron variant). None of the mutations reduced ACE2 binding and cell-cell fusion although several modulated the efficiency of host cell entry. The effects of mutation S686G on viral entry were cell-type dependent and could be linked to the availability of cathepsin L for S protein activation. These results show that polymorphisms at the S1/S2 site can modulate S protein processing and host cell entry.

## Introduction

The pandemic spread of severe acute respiratory syndrome coronavirus type 2 (SARS-CoV-2), the causative agent of coronavirus disease 2019 (COVID-19), was so far associated with over 346 million diagnosed cases and more than 5.5 million deaths as of January 23, 2022 [1]. The viral envelope glycoprotein spike (S) mediates SARS-CoV-2 entry into target cells. For this, the S protein first binds to the cellular receptor angiotensin converting enzyme 2 (ACE2) via its S1 subunit [2, 3]. Subsequently, the S2 subunit fuses the viral membrane with a host cell membrane to allow delivery of the viral genome into the host cell cytoplasm [4]. For efficient entry into lung cells, the S protein requires cleavage at the S1/S2 site [5–8], which is located within an extended loop at the interface of the S1 and S2 subunit and is characterized by the presence of a multibasic motif that is not found in closely related coronaviruses from bats and pangolins [6]. S protein cleavage at the S1/S2 site is carried out by the cellular protease furin [5–7] and efficient cleavage at the S1/S2 site might be required for immune evasion [9] and is a determinant of viral pathogenicity and transmissibility [5–8, 10–15].

Compared to other RNA viruses, coronaviruses are genetically more stable due to a proof-reading activity of the viral polymerase [16]. Nevertheless, mutations in SARS-CoV-2 have been detected and viruses with a D614G exchange became dominant early in the pandemic [17]. The D614G exchange increases the percentage of S proteins present in the “open” conformation required for efficient ACE2 binding and viruses bearing this exchange show accelerated transmission kinetics in animal models [18–22]. Subsequently, a SARS-CoV-2 variant harbouring a distinct set of mutations in the S protein became dominant in several countries, including the United Kingdom (variant B.1.1.7 also termed Alpha variant). Thereafter, variants B.1.351 (Beta variant) and P.1 (Gamma variant) emerged, which efficiently evade neutralisation by antibodies used for therapy or induced upon infection or vaccination [23, 24]. These viruses dominated locally but did not become predominant on a global level. In contrast, variant B.1.617.2 (Delta variant), which emerged in India in spring 2021, spread globally and became dominant in many countries [25–28]. At present, variant B.1.1.529 (Omicron variant), which was first detected in Botswana, South Africa and Hong Kong in November 2021, is displacing B.1.617.2 on a global level [29, 30]. This virus is highly mutated, with more than 30 amino acid changes in the spike protein, and evades antibody-mediated neutralization with unprecedented efficiency [31–34].

Compared to B.1.351 and P.1, evasion from antibody-mediated neutralization by B.1.617.2 and particularly B.1.1.7 is lower [24, 26–28, 35–42] and therefore unlikely the only factor responsible for the rapid spread of these variants. In fact, a constantly emerging body of evidence indicates that the B.1.1.7 and likely also B.1.617.2 variants exhibit increased transmissibility, potentially because of infected patients shedding more virus and being infectious for a longer period [43–50]. Therefore, it is of interest to identify viral and cellular factors that promote viral spread in the upper respiratory tract and thus transmissibility of SARS-CoV-2. Since the S1/S2 site in the viral spike protein is a determinant of transmissibility [12, 13], we asked whether naturally occurring mutations within or close to this site impact S protein cleavage and S protein-driven cell-cell and virus-cell fusion.

## Material and methods

### Cell culture

293T (human embryonic kidney) and Vero cells (African green monkey kidney, kindly provided by Andrea Maisner) were cultured in Dulbecco’s modified Eagle’s medium (DMEM; PAN Biotech) supplemented with 10% fetal bovine serum (FBS; PAN Biotech) and 1% penicillin and streptomycin (pen/strep) from a 100x stock solution (PAN Biotech). Calu-3 (human lung, kindly provided by Stephan Ludwig) and Caco-2 (colon epithelial) cells were cultured in Minimum Essential Media (MEM, Life Technologies) supplemented with 10% FBS, 1% pen/strep, 1% sodium pyruvate (Thermo Fisher Scientific) and 1% non-essential amino acids (PAA). A549 (human lung) cells stably expressing ACE2 (A549-ACE2) [24] were cultivated in DMEM/F-12 Medium with Nutrient Mix (ThermoFisher Scientific) supplemented with 10% FBS and 1% pen/strep. Calu-3 cells stably expressing cathepsin L (CTSL, Gene bank NM_001382757) (Calu-3 (CTSL)) were generated using retroviral transduction and cultured in MEM medium [51]. Culture medium for A549-ACE2 and Calu-3 (CTSL) cells was supplemented with 1 μg/ml puromycin. The cell lines were cultivated at 37 °C and 5% CO_2_ in humidified atmosphere. 293T cells were either transfected by calcium phosphate precipitation or using polyethylenimine (PEI; Polysciences).

### Plasmids

We used previously described plasmids, pCAGGS-DsRed [2], pCAGGS-VSV-G [52], pCG1-SARS-2-SΔ18 [2], pCG1-SARS-2-S-HA [2], pCG1-sol-ACE2-Fc [24], pCG1-ACE2 [53], pCAGGS-TMPRSS2 [54], pCG1-SARS-2-SΔ18 (ΔS1/S2) [6], pGal4-TurboGFP-Luc and pVp16-Gal4 [55]. Expression plasmids for SARS-CoV-2 S mutants were constructed by inserting the respective mutations into the wildtype SARS-2-S sequence (Wuhan-1 isolate; hCoV-19/Wuhan/WH01/2019, GISAID accession ID: EPI_ISL_406798) via overlap extension PCR. using overlapping primers harbouring the desired mutations and plasmids pCG1_SARS-2-S-HA (C-terminal HA epitope tag) or pCG1_SARS-2-S-Δ18 (C-terminal truncation of 18 amino acid residues) as template (primer sequences are available upon request). Subsequently, the open reading frames were inserted into the pCG1 plasmid (kindly provided by Roberto Cattaneo) using restriction sites BamHI and XbaI.

### Cell-cell fusion assay

The cell-cell fusion assay has been carried out as described [55]. 293T target cells were seeded in 48-well plates at a cell density of 40,000 cells/well and transfected with Gal4-TurboGFP-luciferase reporter plasmid as well as expression plasmids for ACE2 alone or in combination with TMPRSS2 in an ACE2/TMPRSS2 ratio of 4:1 using polyethylenimine. 293T effector cells were seeded in 6-well plates at 70 to 80% confluence and cotransfected with the VP16-Gal4 expression plasmid and expression plasmid for wildtype (WT) SARS-CoV-2 S or the respective SARS-CoV-2 S mutant. At 24 h post transfection, effector cells were mechanically detached and added to the target cells in a ratio of 1:1. After 24 h, luciferase activity was measured in cell lysates using the Beetle-Juice luciferase assay (PJK; Biotech) and a BioTek Synergy 2 plate reader according to the manufacturer’s instructions.

### Production of VSV pseudoparticles (VSVpp)

We generated VSV pseudoparticles (VSVpp) as described previously [56]. Briefly, 293T cells were transfected with expression plasmid for WT or mutant SARS-CoV-2 S or plasmid encoding DsRed (negative control). At 24 h posttransfection, cells were inoculated with VSV*ΔG FLuc [57] (kindly provided by Gert Zimmer) for 1 h at 37 °C. Next, the inoculum was removed, the cells were washed once with PBS, and DMEM medium containing an anti-VSV-G antibody (produced in I1 hybridoma cells, ATCC CRL-2700) was added to all cells except for those transfected with VSV-G expression vector (these cells received medium without antibody). The cells were further incubated for 24 h before the VSVpp-containing supernatant was harvested, clarified by centrifugation at 4,000 × g for 5 min, and either used directly or stored at −80 °C.

### Transduction of target cells

For transduction experiments, target cells were seeded in 96-well plates 24 h prior to transduction. For transduction, the culture medium was aspirated, and equal volumes of VSV pseudotypes were added to the cells. At 16–18 h posttransduction, transduction efficiency was quantified by measuring the virus encoded firefly luciferase (fLuc) activity in cell lysates using a commercial kit (Beetle-Juice; PJK) and a Hidex Sense plate luminometer (Hidex).

### Immunoblot

To investigate S protein cleavage and particle incorporation, VSV pseudotypes bearing WT or mutant SARS-CoV-2 S with a C-terminal HA antigenic tag were concentrated by centrifugation (13,300 rpm, 90 min, 4 °C) through a sucrose cushion (20% w/v sucrose) and subsequently lysed in 2x SDS-sample buffer (0.03 M Tris-HCl, 10% glycerol, 2% SDS, 5% beta-mercaptoethanol, 0.2% bromophenol blue, 1 mM EDTA) by incubation at 96 °C for 10 min. After SDS-PAGE, proteins were blotted onto nitrocellulose membranes (Hartenstein) and membranes were blocked for 30 min in PBS-T (PBS containing 0.5% Tween 20) containing 5% skim milk. After blocking, membranes were incubated with primary antibodies against the HA tag (1:1,000, mouse, Sigma-Aldrich) or VSV-M (1:1,000, mouse, Kerafast) overnight. For detection of antibody binding, we used HRP-conjugated anti-mouse secondary antibody (1:2,000, Dianova).

To investigate CTSL expression in stably transduced Calu-3 (CTSL) cells, cell lysates were prepared by incubating parental Calu-3 or Calu-3 (CTSL) cells with 2x SDS-sample buffer for 15 min at room temperature followed by incubation at 96 °C for 10 min. Next, lysates were subjected to SDS-PAGE and blotted onto nitrocellulose membranes. Following blocking, membranes were probed with primary antibodies against the cMYC tag (undiluted supernatant from anti cMYC antibody [clone 9E10]-expressing hybridoma cells, mouse) or beta-actin (ACTB, 1:1,000, mouse, Sigma-Aldrich) overnight. For detection of antibody binding, we used HRP-conjugated anti-mouse secondary antibody (1:2,000, Dianova).

All antibodies were diluted in PBS-T containing 5% skim milk and after each antibody incubation blots were washed three times for 10 min with PBS-T. Immunoblots were developed using a self-made chemiluminescence solution (0.1 M Tris-HCl [pH 8.6], 250 μg/ml luminol, 0.1 mg/ml para-hydroxycoumaric acid, 0.3% hydrogen peroxide) in combination with the ChemoCam imaging system and the ChemoStar Professional software (Intas Science Imaging Instruments).

### Production of soluble ACE2

293T cells were seeded in 6-well plates and transfected with expression plasmid for soluble ACE2-Fc. After overnight incubation, the medium was replaced and the cells further incubated for 36 h before the supernatant was collected and centrifuged to remove cell debris. Further, the culture supernatant was 100-fold concentrated using a Vivaspin protein concentrator column (molecular weight cut-off 30 kDa; Sartorius). All centrifugation steps were done at 4,000 × g, 4 °C. The concentrated soluble ACE2-Fc was aliquoted and stored at −80 °C for further use.

### Binding of soluble ACE2-Fc to S protein

293T cells were seeded in 6-well plates and transfected with expression plasmid for WT or mutant SARS-CoV-2 S. Untransfected 293T cells served as a negative control. At 24 h posttransfection, the medium was replaced by fresh medium. At 48 h posttransfection, the culture medium was removed and cells were resuspended in PBS and transferred into 1.5 ml reaction tubes before being pelleted by centrifugation (centrifugation steps were carried out at room temperature at 600 x g for 5 min). Subsequently, the supernatant was aspirated and the cells were washed with PBS containing 1% BSA (PBS-B) and pelleted by centrifugation. Next, the supernatant was removed and cell pellets were resuspended in 250 μl PBS-B containing soluble ACE2-Fc (1:100) and rotated for 60 min at 4 °C using a Rotospin test tube rotator disk (IKA). Following incubation, cells were pelleted, resuspended in 250 μl PBS-B containing anti-human AlexaFlour-488-conjugated antibody (1:200; Thermo Fisher Scientific) and rotated again for 60 min at 4 °C. Finally, the cells washed with PBS-B, fixed by incubation in 4% paraformaldehyde solution for 30 min at room temperature, washed again and resuspended in PBS-B before being subjected to flow cytometric analysis using a LSR II flow cytometer and the FACS diva software (BD Biosciences). Data were further analyzed using the FCS express 4 Flow research software (De Novo software) in order to determine the geometric mean channel fluorescence.

### Protein models

The full-length S protein sequence of the SARS-CoV-2 WH01 isolate (GISAID Accession identifier: EPI_ISL_406798) was modelled on the published crystal structure PDB: 6XR8 [58] using the SWISS-MODEL tool (https://swissmodel.expasy.org/interactive) and protein models were further visualized and colored employing the YASARA (http://www.yasara.org/index.html) and UCSF Chimera (version 1.14, developed by the Resource 406 for Biocomputing, Visualization, and Informatics at the University of California, San Francisco) software packages. As the crystal structure PDB: 6XR8 lacks the structural information for residues Q677-A688 of the extended S1/S2 loop, the corresponding structure was computationally reconstructed (SWISS-MODEL tool).

### Statistical analysis

We performed two-tailed Student’s t-test with Welch’s correction using the GraphPad Prism 7 software package (version 7.03; GraphPad Software Inc). Only *p* values of 0.05 or lower were considered statistically significant (*p* > 0.05 [ns, not significant], *p* ≤ 0.05 [*], *p* ≤ 0.01 [**], *p* ≤ 0.005 [***]).

## Results

### Mutations P681H, P681L, A684S, S686G and V687L reduce S protein cleavage

**Fig 1:**
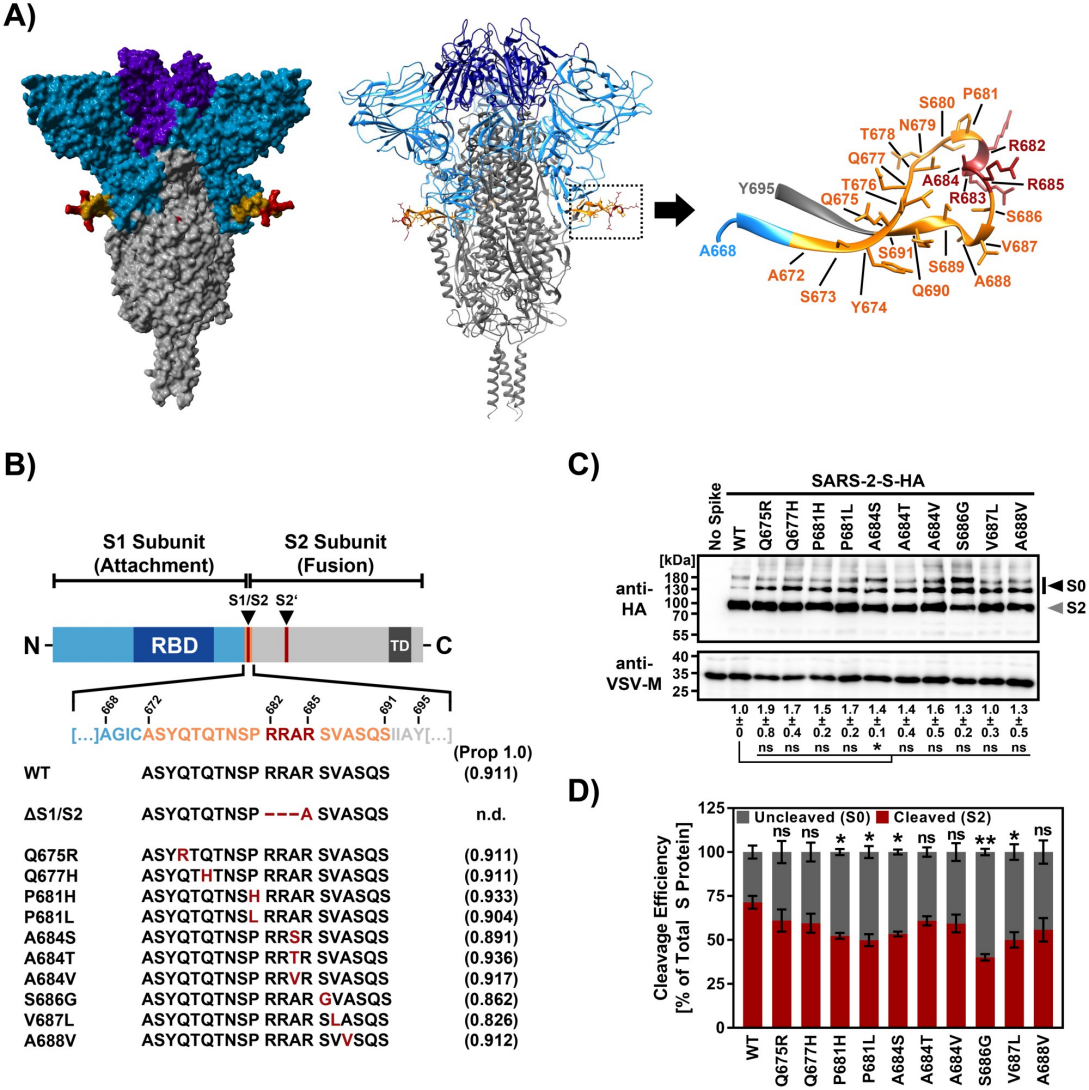
Mutations at S1/S2 site reduce SARS-CoV-2 S cleavage. A) 3D reconstruction of the SARS-CoV-2 S protein trimer. The location of the mutations is highlighted and magnified. Colour code: light blue—S1 subunit with RBD in purple, grey—S2 subunit, orange—cleavage loop encompassing the S1/S2 cleavage site, red—S1/S2 cleavage site. Since the crystal structure PDB: 6XR8 lacks the structural information for residues Q677-A688 of the extended S1/S2 loop, the corresponding structure has been computationally reconstructed. B) Schematic illustration of S protein domain organization. RBD—receptor binding domain, TD—transmembrane domain. The S1/S2 and S2’ cleavage sites are indicated. Mutations located within or adjacent to the S1/S2 site are highlighted. Values in brackets indicate the scores by furin cleavage prediction (ProP 1.0; n.d., not determinable) C) Incorporation of SARS-CoV-2 S proteins into VSV particles. Pseudotyped particles harbouring the indicated S proteins equipped with a C-terminal HA antigenic tag were subjected to immunoblot analysis, using anti-HA antibody. Black and grey filled arrows indicate uncleaved precursor SARS-CoV-2 S (S0) and S2, respectively. Detection of VSV-M served as a loading control. Shown is a representative immunoblot from three independent experiments. Further, total S protein levels in particles were quantified. For this, S protein signals were first corrected with respect to the corresponding VSV-M signals and subsequently normalized (WT SARS-CoV-2 = 1). The average data from three experiments (+/- the standard error of the mean, SEM) are shown below the immunoblot. D) Quantification of cleavage efficiency. For each S protein total S protein signals (S0 + S2) were set as 100% and the relative proportion of the individual S0 and S2 signals were calculated. Displayed are the average data from three independent experiments. Error bars represent the SEM. Statistical significance was analysed by two-tailed Student’s t-test with Welch’s correction (p > 0.05, not significant [ns], p ≤ 0.05, *; p ≤ 0.01, **). WT = wildtype.

SARS-CoV-2 S protein amino acid residues 672 to 691 encompass the extended loop harbouring the S1/S2 cleavage site and are frequently polymorphic [59] (Fig 1A). To investigate the potential impact of naturally-occurring amino acid exchanges within this region on SARS-CoV-2 host cell entry, we selected a total of ten mutations for detailed analysis (Fig 1B and S1 Fig). Selection of mutations was based on frequency of occurrence in SARS-CoV-2 sequences deposited in the NCBI (National Center for Biotechnology Information) database (as of October 2020). The respective mutations were inserted into expression plasmids for the S protein of the SARS-CoV-2 Wuhan-1 isolate (hCoV-19/Wuhan/WH01/2019, GISAID accession ID: EPI_ISL_406798). Three mutations are located within the S1/S2 cleavage motif 682-RRAR-685 (A684S, A684T or A684V), while two mutations affect the proline residue that directly precedes the cleavage motif (P681H, P681L) and one of them is present in the B.1.1.7 variant and B.1.1.529 variants (P681H). The remaining mutations under study include Q675R, Q677H, S686G, V687L and A688V (Fig 1B). A prediction of the likelihood of S protein cleavage by furin and other proprotein convertases was carried out using the ProP 1.0 prediction tool (http://www.cbs.dtu.dk/services/ProP/) but did not reveal fundamental differences between WT and mutant sequences (Fig 1B). The only exception was an artificial mutant spike protein in which the multibasic cleavage motif had been replaced by a single alanine residue (ΔS1/S2).

We first assessed whether the mutations alter S protein incorporation into the vesicular stomatitis virus (VSV) pseudotype particles used for the study of S protein-driven entry and S protein cleavage at the S1/S2 site. For this, we employed S proteins harbouring a C-terminal antigenic HA tag and immunoblot analyses (Fig 1C and S2 Fig). Detection of the VSV matrix protein (VSV-M) served as internal reference. We found that all S protein mutants were robustly incorporated into particles and no significant differences in S protein incorporation between WT and mutant SARS-CoV-2 S were observed, except for mutant A684S, for which particle incorporation was slightly (factor: 1.4 +/- 0.1) increased (Fig 1C). The particles harboured mainly S protein processed at the S1/S2 site, as evidenced by a prominent ∼90 kDa band corresponding to the S2 subunit, as expected [2, 60]. In addition, much less prominent bands corresponding to uncleaved S protein (S0) were detected. Quantification of the S0 and S2 signals from multiple experiments revealed that none of the ten mutations tested increased S protein cleavage at the S1/S2 site (Fig 1C and 1D).

In contrast, five mutations (P681H [cleavage efficiency: 52.3% +/- 1.7%], P681L [49.9% +/- 3.4%], A684S [53.4% +/- 1.4%], S686G [40.1% +/- 1.8%] and V687L [50.0% +/- 4.4%]) were found to cause a significant decrease (∼1.34- to 1.80-fold) in S protein cleavage compared to the WT SARS-CoV-2 S (71.4% +/- 3.7%) (Fig 1C and 1D). Thus, P681H and several other naturally-occurring polymorphisms can modulate S protein cleavage efficiency.

### Mutation S686G slightly augments ACE2 binding

**Fig 2:**
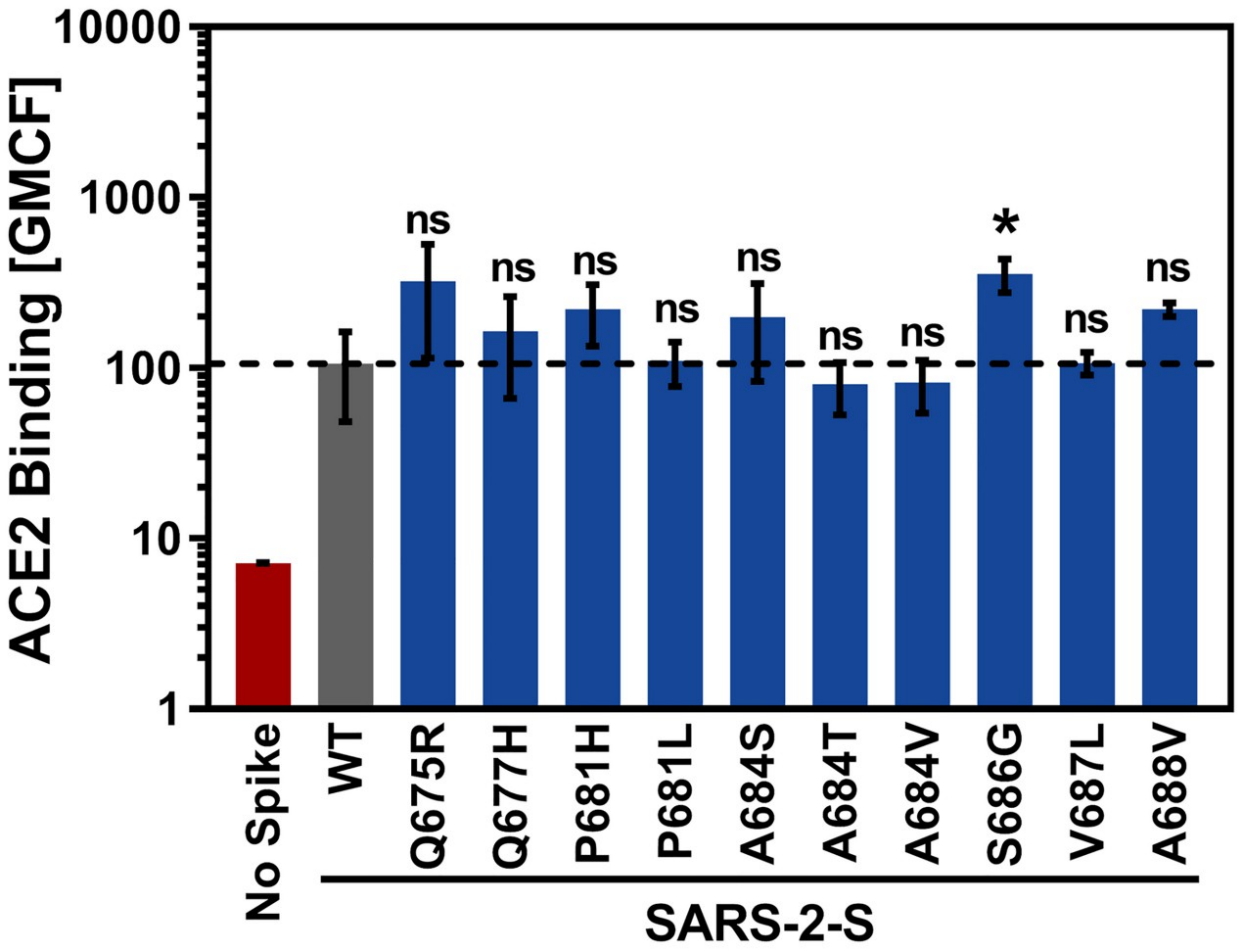
Mutations at the S1/S2 site have little impact on soluble ACE2-Fc binding. 293T cells expressing the indicated S protein mutants were incubated with soluble ACE2-Fc and binding was detected using an Alexa488-coupled secondary antibody and flow cytometry. Cells that did not express S protein were used as negative control. The average geometric mean channel fluorescence (GMCF) of three independent experiments is shown. Error bars represent the standard deviation (SD). Statistical significance was analysed by two-tailed Student’s t-test with Welch’s correction (p > 0.05, not significant [ns], p ≤ 0.05, *). WT = wildtype.

Binding to the cellular receptor ACE2 is required for infectious entry and any effect on S protein-driven cell-cell or virus-cell fusion by the mutations under study might simply reflect altered ACE2 binding. We addressed this possibility by analysing binding of soluble ACE2 to cells expressing WT or mutant SARS-CoV-2 S protein. For this, the ACE2 ectodomain fused to the Fc portion of human immunoglobulin was used. None of the tested mutant S proteins displayed reduced ability to bind to ACE2 compared to WT SARS-CoV-2 S and for most mutant S proteins no significant changes in ACE2 binding efficiency were observed (Fig 2). The only exception was mutation S686G, which caused a moderate but significant increase in ACE2 binding (Fig 2). In sum, the mutations studied here did not compromise ACE2 binding and one slightly augmented binding.

### S686G modulates SARS-CoV-2 S-driven entry in a cell line-dependent manner

**Fig 3:**
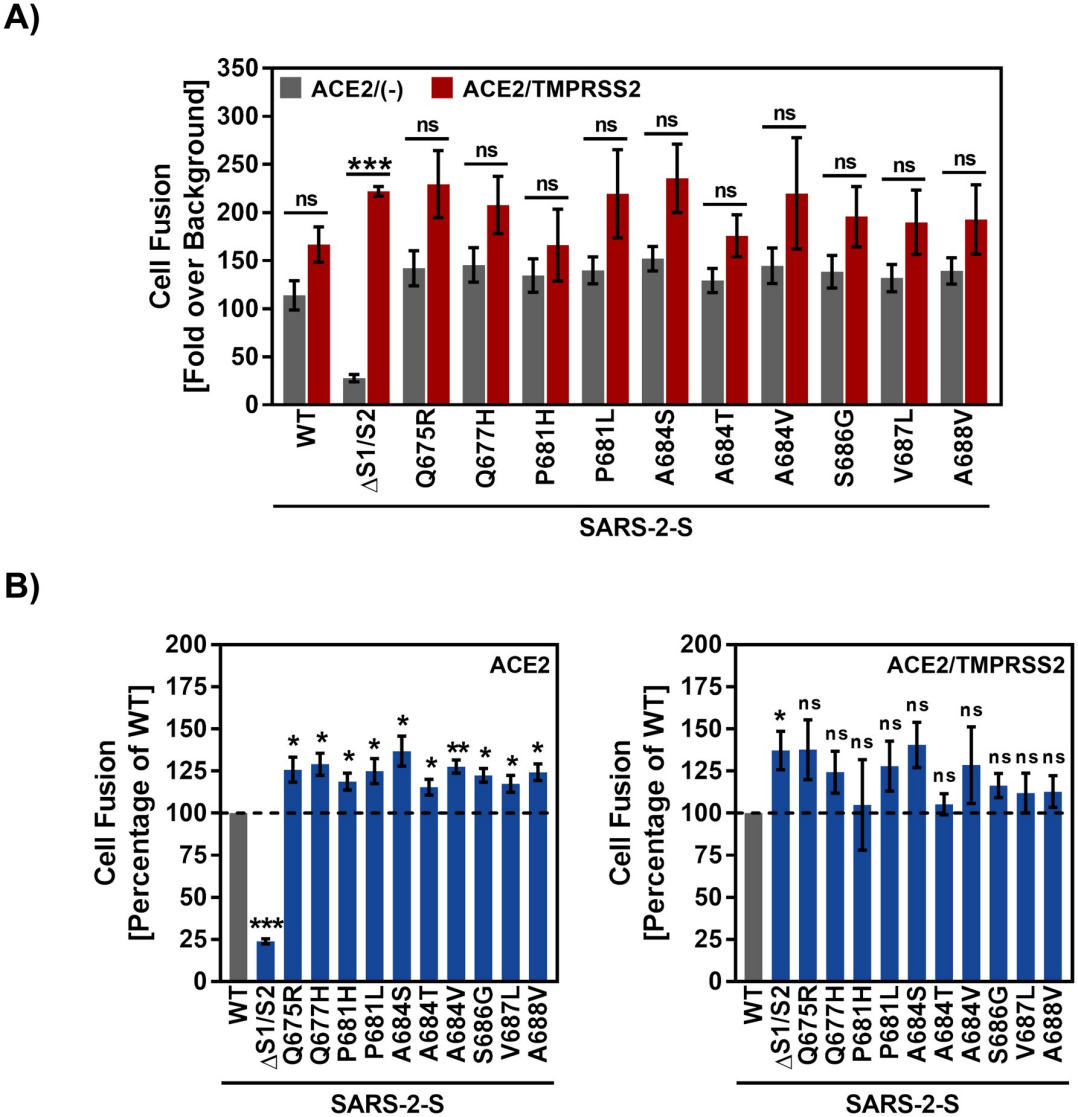
SARS-CoV-2 mutants mediate robust cell-cell fusion. A) Effector cells cotransfected with Vp16-Gal4 transactivator plasmid and pCG1 empty vector (negative control) or expression plasmids for the indicated S proteins were co-cultured with target cells cotransfected with Gal4-TurboGFP-Luc reporter plasmid and either ACE2 expression plasmid alone or in combination with TMPRSS2 expression plasmid. At 24 h post transfection, luciferase activity in cell lysates was measured. Displayed are the average data from four independent experiments (each performed with biological triplicates) where cell-cell fusion was either normalized against the assay background (A; fold over background, set as 1) or against WT SARS-CoV-2 S (B; percentage, set as 100%). Error bars represent the SEM. Statistical significance was analysed by two-tailed Student’s t-test with Welch’s correction (p > 0.05, not significant [ns], p ≤ 0.05, *; p ≤ 0.01, **; p ≤ 0.001, ***). WT = wildtype.

The SARS-CoV-2 S protein can drive cell-cell fusion, resulting in the formation of syncytia, and this activity is believed to contribute to COVID-19 pathogenesis [61, 62]. We employed a previously described assay to measure cell-cell fusion driven by SARS-CoV-2 S WT and mutants ([55]; see Methods). The WT S protein expressed on 293T effector cells drove fusion with 293T target cells transfected to express ACE2 and fusion was moderately increased upon coexpression of ACE2 with TMPRSS2 (Fig 3A and 3B), as expected [6]. In contrast, deletion of the S1/S2 site markedly reduced fusion with target cells transfected to express ACE2 in keeping with published data [6] and this phenotype was rescued upon coexpression of TMPRSS2 (Fig 3A and 3B), likely due to conditions of overexpression. Finally, all S protein mutants facilitated fusion with ACE2 or ACE2/TMPRSS2 expressing cells with at least the same efficiency as SARS-CoV-2 S WT (Fig 3B). In sum, the mutations analysed were compatible with efficient cell-cell fusion.

We next investigated whether the mutations modulated virus-cell fusion. For this, we employed two human lung cell lines, Calu-3 and A549, as well as the human colon cell line Caco-2 and the African green monkey kidney cell line Vero as targets. While Vero, Calu-3 and Caco-2 have been previously shown to be highly susceptible to SARS-CoV-2 S-driven cell entry, A549 cells were only moderately susceptible most likely due to low ACE2 expression [2]. Therefore, we employed A549 cells that were modified to overexpress ACE2 in order to enhance their susceptibility to SARS-CoV-2 S-driven entry. After engaging ACE2, the SARS-CoV-2 S protein further requires to be activated by cellular proteases in order to drive fusion of the viral and cellular membranes and depending on the availability of such proteases, fusion can either take place at the plasma membrane (activation via TMPRSS2 and related serine proteases) or within endosomal vesicles following endocytosis (activation via cathepsin L, CTSL) [2, 6]. We thus included mainly cell lines for which the protease availability and thus entry pathway is clearly defined: Calu-3 and Caco-2 cells, which express endogenous TMPRSS2, and Vero cells that lack TMPRSS2 expression and only allow for S protein activation via CTSL [2, 6].

**Fig 4:**
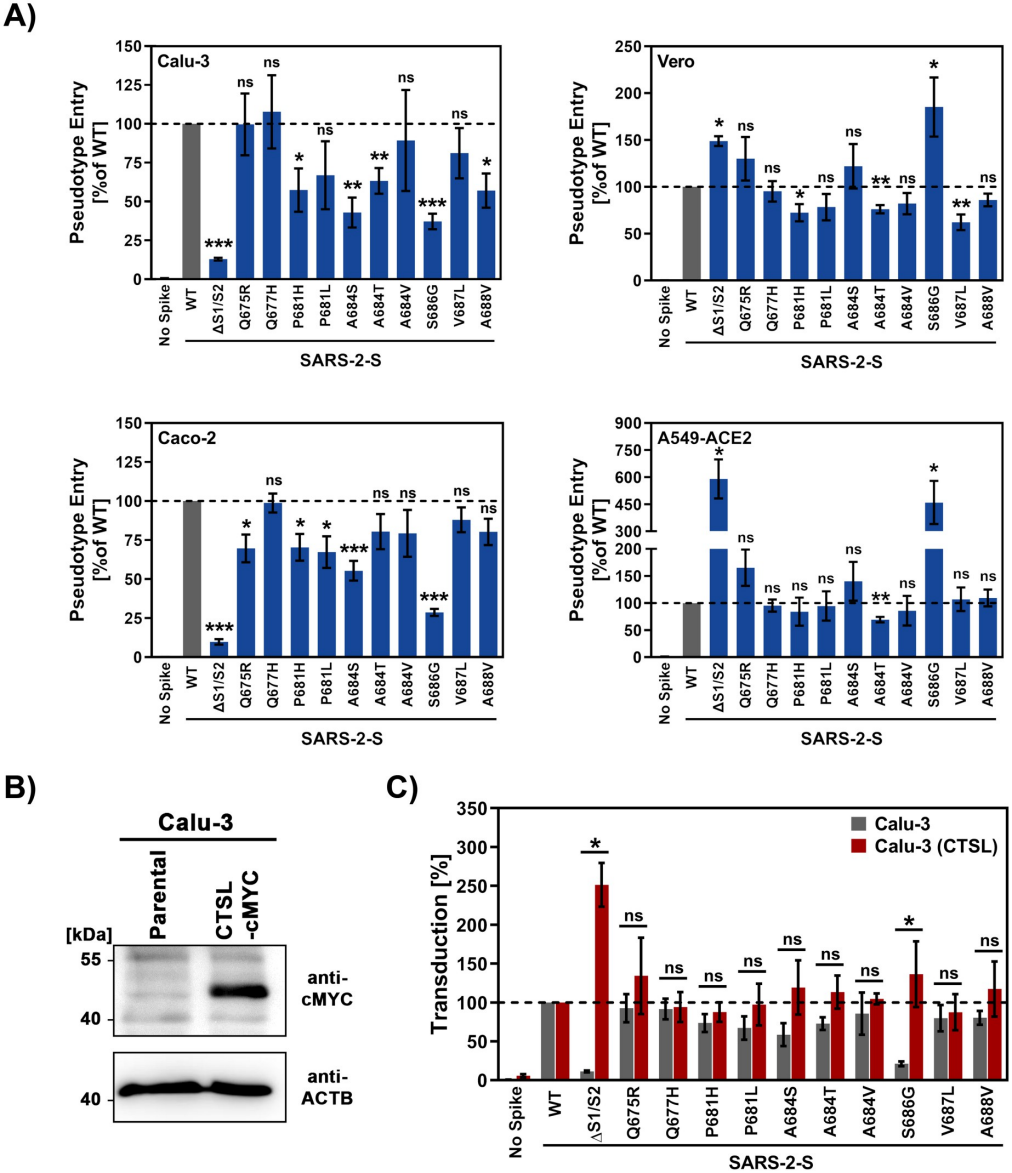
S686G modulates SARS-CoV-2 S-driven entry in a cell line-dependent manner. A) The indicated cell lines were inoculated with equal volumes of pseudotyped particles bearing the indicated S proteins or no S protein (negative control). Transduction efficiency was quantified by measuring virus encoded luciferase activity in cell lysates at 16–20 h post transduction. The average from six independent experiments is shown. Error bars represent SEM. B) Stable overexpression of cathepsin L (CTSL) in Calu-3 cells. Cell lysates of parental Calu-3 cells and Calu-3 cells stably expressing CTSL harbouring a C-terminal cMYC epitope tag (CTSL-cMYC) were subjected to immunoblot analysis using anti-cMYC antibody. Detection of beta-actin (ACTB) served as a loading control. Shown is a representative immunoblot from two independent experiments. C) The experiment was carried out as described for panel A but entry into Calu-3 WT and Calu-3 cells stably expressing CTSL was analysed. Statistical significance was analysed by two-tailed Student’s t-test with Welch’s correction (p > 0.05, not significant [ns], p ≤ 0.05, *; p ≤ 0.01, **; p ≤ 0.001, ***). WT = wildtype.

All S protein mutants mediated entry into the tested cell lines although with different efficiency when compared to WT SARS-CoV-2 S (Fig 4A). Mutants Q675R, Q677H, P681H, P681L, A684S, A684T, A684V and V687L facilitated entry into all cell lines tested with comparable or up to two-fold enhanced/decreased efficiency compared to WT SARS-CoV-2 S (Fig 4A). Out of the ten S protein mutants tested, one (S686G) showed variable and cell line-dependent phenotypes when compared to WT SARS-CoV-2 S. Mutation S686G attenuated entry into Calu-3 and Caco-2 cells by roughly 2.5- and 3.5-fold, respectively, while entry into Vero and A549-ACE2 cells was enhanced by 1.8- and 4.5-fold, respectively. A similar phenotype was further observed for an artificially designed S protein that lacks the multibasic S1/S2 motif (mutant ΔS1/S2, Fig 4A).

### Exogenous cathepsin L expression rescues Calu-3 cell entry of SARS-CoV-2 S mutant S686G

Finally, we sought to obtain initial insights into why mutation S686G reduced viral entry into Calu-3 cells. Entry into this cell line is mainly TMPRSS2 dependent [6], potentially due to insufficient expression of CTSL [63]. Therefore, we investigated whether overexpression of CTSL in Calu-3 cells increases the efficiency of entry of certain S protein mutants. Immunoblot analysis revealed robust CTSL expression in Calu-3 (CTSL) cells (Fig 4B and S2 Fig). Overexpression of CTSL did not significantly affect entry driven by most S protein mutants when compared to WT SARS-CoV-2 S (Fig 4C). Strikingly, entry driven by the S protein mutant lacking the S1/S2 site was markedly enhanced upon directed expression of CTSL and even exceled entry driven by WT S protein. Also, entry of mutant S686G was significantly augmented by directed CTSL expression, suggesting that insufficient endogenous CTSL levels limit Calu-3 cell entry of this mutant. These results suggest that CTSL levels can limit S protein-driven entry into Calu-3 cells, an effect that is particularly prominent in the absence of an intact S1/S2 site.

## Discussion

We analysed several naturally-occurring amino acid variations that have been found in the extended loop harboring the SARS-CoV-2 S S1/S2 cleavage site. A total of five mutations (P681H, P681L, A684S, S686G, V687L) were found to be associated with reduced S protein cleavage, with mutation S686G showing the lowest level of S protein cleavage. Except for mutation S686G, which moderately increased S protein binding to soluble ACE2, no mutation was associated with changes in receptor binding, in keeping with the mutations being located outside the receptor-binding domain (RBD). Why mutant S686G showed enhanced interaction with ACE2 is at present unclear but might be due to its RBD adopting a conformation that may favour ACE2 binding, which would be a similar effect as reported for mutation D614G that is also located outside of the RBD [19]. With respect to the ability of the SARS-CoV-2 S protein to drive fusion of S protein expressing cells with neighbouring cells, we observed that all mutations, including S686G, were compatible with robust spike protein-induced cell-cell fusion. This observation is different to the results reported by Lamers and colleagues, who found that cell-cell fusion driven by mutant S686G is less efficient as compared to WT S [64]. One can speculate that differences in the fusion assays employed by Lamers et al. and us may be the reason for the different findings. For example, the assay used by Lamers et al. uses Vero E6, Vero E6-TMPRSS2 and Calu-3 cells in a GFP complementation assay, while we employed 293T cells transfected to overexpress either ACE2 alone or in combination with TMPRRS2 and a luciferase-based reporter. Thus, high ACE2 expression levels on our 293T cells may compensate for the reduced cell-cell fusion ability by SARS-CoV-2 S bearing mutation S686G.

With respect to SARS-CoV-2 S protein-driven cell entry, exchange S686G was associated with reduced entry efficiency for the cell lines Calu-3 and Caco-2 while entry into Vero and A549-ACE2 cells was significantly augmented compared to WT SARS-CoV-2 S. SARS-CoV-2 entry into Calu-3 and Caco-2 cells depends on S protein activation by TMPRSS2, which in turn requires S protein cleavage at the S1/S2 site by furin [2, 6]. The observation that mutation S686G reduced S protein cleavage at S1/S2 with the highest efficiency of all mutations studied indicates that decreased Calu-3 and Caco-2 cell entry of mutant S686G may have been due to reduced TMPRSS2 usage, which is in line with the observations by Lamers and colleagues [64]. In keeping with the previously suggested scenario that availability of endogenous CTSL may limit CTSL-dependent entry into Calu-3 cells [63, 65], directed expression of CTSL increased Calu-3 cell entry of mutant S686G and mutant ΔS1/S2.

It is interesting to note that variant of concern B.1.617.2 (Delta variant), harbours exchange P681R, which was recently shown to increase S protein cleavage and cell-cell fusion, demonstrating that naturally occurring mutations at the S1/S2 site can augment S protein cleavability and activity [66, 67]. In contrast, Lubinski and colleagues [68] and the present study demonstrate that P681H, which is found in the S proteins of the B.1.1.7 and B.1.1.529, does not increase S protein cleavage as well as S protein-driven cell-cell and virus-cell fusion.

Collectively, our results suggest that naturally-occurring mutations located within the extended S1/S2 loop may modulate SARS-CoV-2 infectivity, in part by altering protease usage. However, it needs to be noted that the mutations were studied in the context of WT S protein and not the S proteins from which they were derived, which precluded detection of effects dependent on the sequence context. Further, we utilized a replication-deficient vesicular stomatitis virus pseudotyped with WT or mutant SARS-CoV-2 S proteins to investigate entry into cell lines. Although it is widely accepted that such pseudotyped particles faithfully reflect the entry process of authentic SARS-CoV-2, our data await formal confirmation with replication-competent SARS-CoV-2 and primary respiratory cell cultures.

## Supporting information

S1 Fig Ribbon models of the extended S1/S2 loop containing the mutations under study. The indicated mutations in the extended S1/S2 loop were introduced using UCSF Chimera (version 1.14). Colour code: blue = residues 668–671 of the S1 subunit that are located upstream of the S1/S2 loop; orange = extended S1/S2 loop (residues 672–691); red = multibasic S1/S2 cleavage site (682-RRAR-685) within the extended S1/S2 loop; grey = residues 692–695 of the S2 subunit that are located downstream of the S1/S2 loop.
(TIF)

S2 Fig Unprocessed images of immunoblots shown in Figs 1C and 4B. (TIF)
