# Supplementary figures and images for: Functional analysis of polymorphisms at the S1/S2 site of SARS-CoV-2 spike protein
Source: PLoS One. 2022 Mar 25;17(3):e0265453. doi: 10.1371/journal.pone.0265453 (PMC8956166; doi:10.1371/journal.pone.0265453)

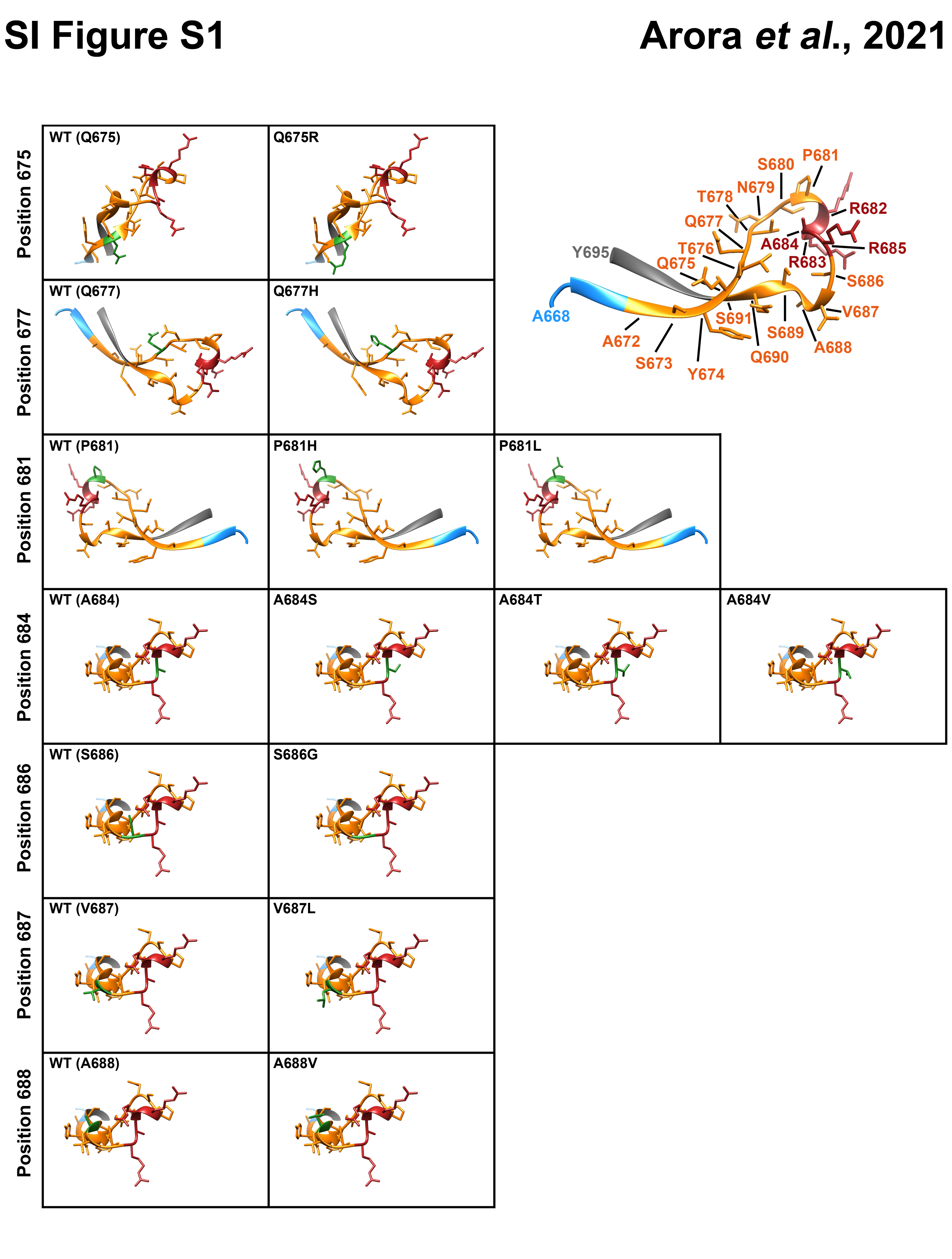

Supplement: S1 Fig — The indicated mutations in the extended S1/S2 loop were introduced using UCSF Chimera (version 1.14). Colour code: blue = residues 668–671 of the S1 subunit that are located upstream of the S1/S2 loop; orange = extended S1/S2 loop (residues 672–691); red = multibasic S1/S2 cleavage site (682-RRAR-685) within the extended S1/S2 loop; grey = residues 692–695 of the S2 subunit that are located downstream of the S1/S2 loop. (TIF) [file pone.0265453.s001.tif]

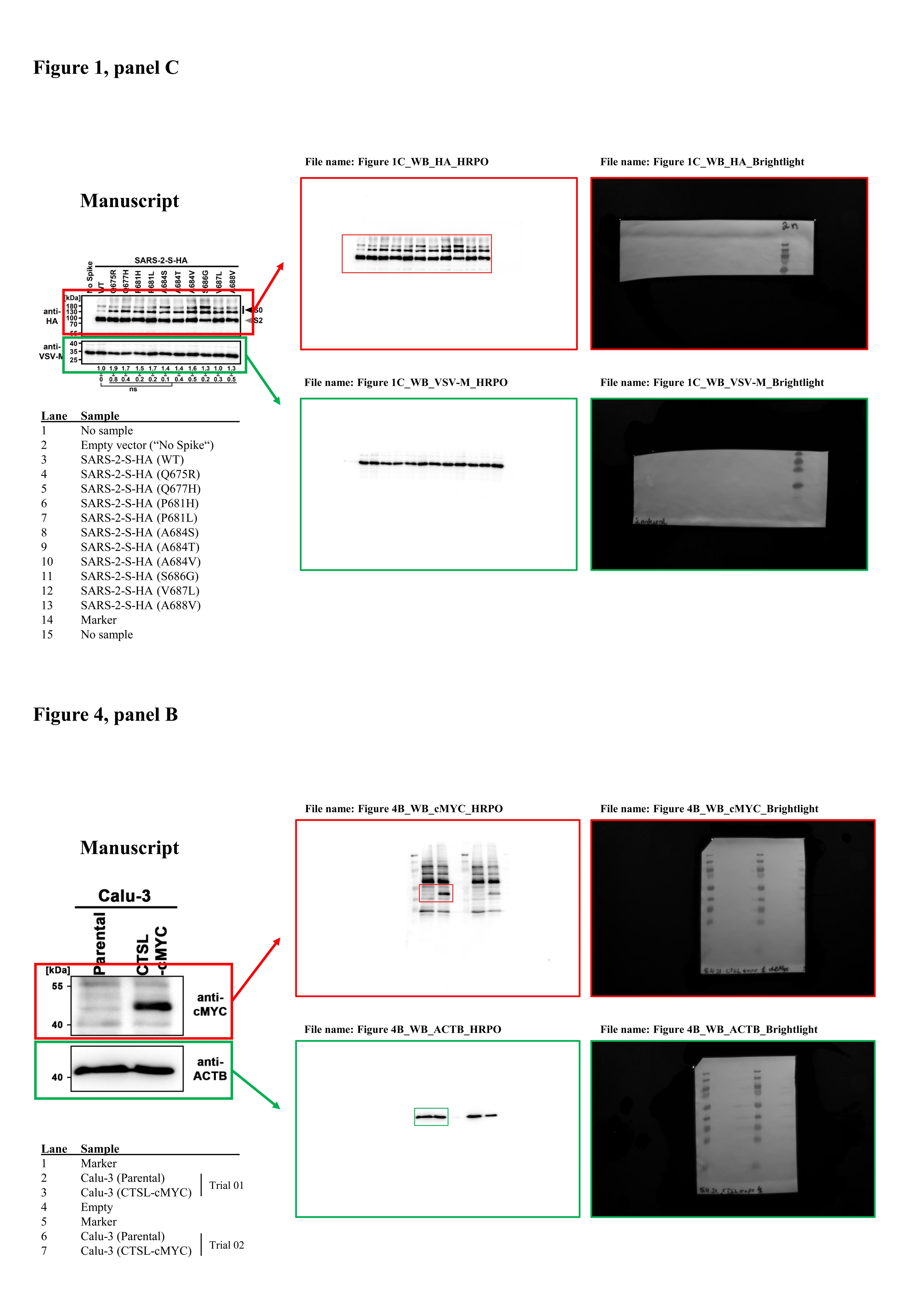

Supplement: S2 Fig — (TIF) [file pone.0265453.s002.tif]
